# Genomic characterization of invasive Streptococcus pneumoniae isolates from a southern Taiwan medical centre, 2015–2023

**DOI:** 10.1099/mgen.0.001730

**Published:** 2026-05-22

**Keywords:** antimicrobial resistance, invasive pneumococcal disease, pneumococcal conjugate vaccine, serotype

## Abstract

Since the introduction of pneumococcal conjugate vaccines, the incidence of invasive pneumococcal disease (IPD) among children has decreased significantly. The distribution of IPD cases has shifted towards other vulnerable populations, including older adults and individuals with pre-existing medical conditions. Alongside this shift, there has also been a concurrent change in pneumococcal serotype distribution. Studies on the distribution of pneumococcal serotypes and antibiotic resistance patterns in the post-pneumococcal conjugate vaccine era, particularly following the coronavirus disease 2019 (COVID-19) pandemic, remain limited in the Asia-Pacific region. This study aims to investigate patient characteristics, pneumococcal serotype and antibiotic resistance profiles predicted by whole-genome sequencing among IPD patients in a public medical centre in southern Taiwan. A total of 61 IPD isolates were collected between 2015 and 2023. Serotype and antibiotic resistance were determined using whole-genome sequencing. The results showed that 47.5% of cases were in patients aged 65 years and older and 34.3% of cases involved patients with comorbid malignancy. The serotypes included in the 13-valent pneumococcal conjugate vaccine (PCV13) comprised 52.5% of the isolates. Overall, 26.2% of isolates were non-susceptible to penicillin (non-meningitis breakpoint). For the PCV13 serotypes, the non-susceptible rate to penicillin (non-meningitis breakpoint) was 43.8%. Phylogenetic analysis suggested recent emergence of a multi-drug resistant serotype34/ST9395 lineage. These findings suggest that the older adult population and those with pre-existing medical conditions, particularly malignancy, could benefit from targeted prevention efforts.

**Impact Statement**

The introduction of the 13-valent pneumococcal conjugate vaccine (PCV13) into childhood immunization programmes has substantially reduced invasive pneumococcal disease (IPD) among children under 5 years of age. However, the burden of IPD has increasingly shifted to adults, particularly those aged 65 years and older. Despite this, PCV13 vaccination for adults remains unfunded in many regions due to fiscal constraints. This study identifies specific adult populations at elevated risk for IPD, providing evidence to inform vaccination policy. These findings support targeted expansion of PCV13 coverage to high-risk adult groups, which may improve disease prevention and optimize public health resource allocation.

## Data Summary

The sequence data of the 61 pneumococcal isolates have been deposited in the National Center for Biotechnology Information under the accession numbers SRX26529323 through SRX26529383.

## Introduction

*Streptococcus pneumoniae* (pneumococcus) is a gram-positive bacterium with over 100 serotypes. Pneumococcal vaccines have been developed to target the most prevalent pneumococcal serotypes and decrease the incidence of pneumococcal diseases. The 23-valent pneumococcal polysaccharide vaccine (PPSV23) includes 23 serotypes of capsular polysaccharides [1]. However, a major limitation of PPSV23 was its inability to stimulate immunity in infants and toddlers under the age of 2 years, a demographic group that is at the highest risk for pneumococcal disease [2]. To address this issue, the seven-valent pneumococcal conjugate vaccine (PCV7) was launched in 2000, specifically targeting the seven most common serotypes implicated in pneumococcal disease [3]. Following the introduction of PCV7, there was a noted serotype replacement, with non-PCV7 serotypes, particularly 19A, emerging on a global scale [4]. To provide broader protection, the 13-valent pneumococcal conjugate vaccine (PCV13) was introduced in 2010, incorporating the serotypes included in PCV7 along with 6 additional emerging serotypes, including 19A. Despite the high effectiveness of PCV13 against invasive pneumococcal disease, serotype replacement driven by vaccine-induced selection pressure continues [5]. This underscores the necessity for continuous surveillance of pneumococcal serotype dynamics to inform the development of next-generation pneumococcal vaccines. The two demographic groups that exhibit the greatest vulnerability to invasive pneumococcal disease (IPD) are children under the age of 5 years and adults aged 65 years and older. Owing to financial constraints within fiscal budgets, routine vaccination programmes typically prioritize children under the age of 5 years. For adults 65 and older, protection is predominantly dependent on indirect herd immunity conferred by vaccinated children. Currently, only a limited number of developed nations extend coverage of PCV13 and/or PPSV23 to adults over the age of 65 [6].

Taiwan implemented PCV13 through a phased strategy. From 2009 to 2014, PCV13 was recommended only for high-risk children under 5 years of age; since 2015, coverage has expanded to all children aged 2 months to 5 years, achieving a 96.8% third-dose uptake by 2023 [7]. In adults, PPSV23 has been provided to those aged ≥75 years since 2008, and sequential PCV13–PPSV23 vaccination was extended to all adults aged ≥65 years in 2024. Following widespread paediatric vaccination, the incidence of IPD declined markedly after 2015, with the incidence rate of IPD decreasing from 17.8/100 000 in 2012 to 5.5/100 000 in 2017 among children aged 0–5 years [8,9]. Furthermore, the incidence of IPD in adults over 70 years of age decreased by 37% from 2012 to 2017, primarily as a consequence of the indirect herd immunity conferred by vaccinated children [9]. Concurrently, serotype replacement was observed, with a shift towards non-PCV13 serotypes [8,11].

Continuous surveillance of serotype substitution and populations at risk for IPD is essential for informing vaccine implementation strategies. Recently, there has been a re-emergence of adult cases of IPD after countries relaxed their nonpharmaceutical interventions to contain the COVID-19 pandemic [12,13]. It is crucial to continuously monitor the epidemiological data pertaining to IPD in the adult demographic group after the successful PCV13 vaccination campaign among paediatric populations. In this study, we identified IPD cases in a public medical centre in southern Taiwan from 2015 to 2023. IPD isolates were characterized by whole-genome sequencing (WGS), which provided comprehensive genetic insights as well as patterns of antibiotic resistance. The findings revealed IPD case characteristics, isolate serotypes and antibiotic resistance patterns through the pandemic period, thereby providing timely data to inform public health policies and vaccine development.

## Methods

### IPD cases and isolates

IPD cases and isolates were identified from 2015 through 2023 in Kaohsiung Veterans General Hospital, Taiwan. This hospital is a governmental tertiary-care facility with 1,477 beds, which mainly serves the two southern administrative divisions (Kaohsiung City and Pingtung County) with a population size of 3.52 million residents. All isolates were collected from patients with IPD admitted to this hospital. The definition of IPD was illness with *S. pneumoniae* cultured from normally sterile body sites, including cerebrospinal fluid, pleural and peritoneal fluids and blood. We defined the years from 2015 through 2019 as the pre-pandemic period and the years 2020 through 2023 as the pandemic period. Micro-organisms cultured from clinical specimens were identified using the VITEK MS system (biomerieux, Marcy-l’Etoile, France). *S. pneumoniae*-positive samples were sub-cultured on trypticase soy agar supplemented with 5% sheep blood and maintained at 35 °C with 5% CO_2_ to recover isolates. IPD isolates were kept at −80 °C in a skimmed milk-trypticase soy-glucose glycerol medium and transported to the Centers for Disease Control and Prevention, Atlanta, USA, for WGS-based strain characterization. Patient demographic information and pre-existing medical conditions were extracted from medical records. The study protocol was approved by the Institutional Review Board of the Kaohsiung Veteran General Hospital (IRB no.:KSVGH25-CT6-15).

### Whole-genome sequencing

Genomic DNA for short-read WGS was extracted using the QIAamp DNA Mini Kit protocol (QIAGEN, Inc., Valencia, CA, USA). Nucleic acid concentration was quantified by an Invitrogen Qubit assay (Thermo Fisher Scientific Inc., Waltham, MA, USA), and samples were sheared using a Covaris M220 or R230 ultrasonicator (Covaris, Inc., Woburn, MA, USA) programmed to generate 500 bp fragments. Libraries were constructed on the SciCloneG3 (PerkinElmer Inc., Waltham, MA, USA) using a TruSeq DNA PCR-Free HT library preparation kit with 96 dual indices (Illumina Inc., San Diego, CA, USA) or a sparQ DNA library kit (Quanta Bio, Beverly, MA) and quantified by a KAPA qPCR library quantification method (Kapa Biosystems Inc., Wilmington, MA, USA). The libraries were sequenced employing the MiSeq instruments and the MiSeq v2 500-cycle or MiSeq v3 600-cycle flow-cell kit (Illumina Inc., San Diego, CA, USA). VelvetOptimiser version 2.2.6 (cite PMID: 18349386) was used to generate a draft whole-genome assembly from WGS reads. Pneumococcal serotype, multilocus sequence type, penicillin-binding protein sequence type and antibiotic genes were annotated using a bioinformatic pipeline as previously described (cite PMID: 27542334). The completeness and contamination metrics of genome assemblies were evaluated by using CheckM version 1.2.4 (cite PMID: 25977477). The 61 draft whole-genome assemblies in this study showed a median completeness of 99.4% (interquartile range, 99.4–99.6%) and a median contamination of 0.29% (interquartile range, 0.24–0.60%).

### Bioinformatic analysis

WGS reads of each IPD isolate were processed and analysed by a validated bioinformatic pipeline to generate a draft genome assembly and to infer strain features, including serotype, multilocus sequence type (ST), penicillin-binding protein (PBP) sequence type and antimicrobial susceptibility (AST) predictions for 18 antibiotics [14]. Briefly, serotype was determined by mapping WGS reads to a custom sequence database to identify the presence of serotype-specific target sequences. ST was determined by using the SRST2 software [15] and the pubMLST database for *S. pneumoniae* [16]. Rather than conducting AST by the broth microdilution method, antimicrobial resistance was predicted using WGS data. Protein sequences in the transpeptidase domains of PBP1a, PBP2b and PBP2x were used to determine the PBP type and were used in a machine-learning algorithm to predict ASTs for *β*-lactam antibiotics [17,18]. To predict ASTs for non-*β*-lactam antibiotics, WGS reads were mapped to a custom resistance gene sequence database. The susceptibility predictions generated minimum inhibitory concentrations (MICs) for each antibiotic, and the MICs were interpreted according to CLSI guidelines [19]. Antibiotic nonsusceptibility is defined as either resistance or intermediate susceptibility to the antibiotic. The bioinformatics pipeline, including databases and scripts, is available at a GitHub repository [14,17, 18]. All WGS data were deposited at the National Center for Biotechnology Information with BioProject number PRJNA1179409.

Phylogenetic analysis was performed for each ST that contained four or more isolates using the kSNP software version 4.1 to generate a parsimony tree based on only core SNPs [20]. The phylogenetic trees were visualized and annotated using the R ggtree package [21].

### Statistical methods

Categorical variables were presented in numbers and proportions of the total numbers. The association between two categorical variables was evaluated by Fisher’s exact test. Continuous variables were presented as mean±sd. Student’s t-test was used to compare the means between two groups for a continuous variable. All tests were two-sided and a *P* value lower than 0.05 was considered statistically significant. *P* values were not adjusted due to the exploratory nature and should be interpreted with caution. All statistical analyses were performed in SAS version 9.4.

## Results

### IPD case features

**Fig. 1.**
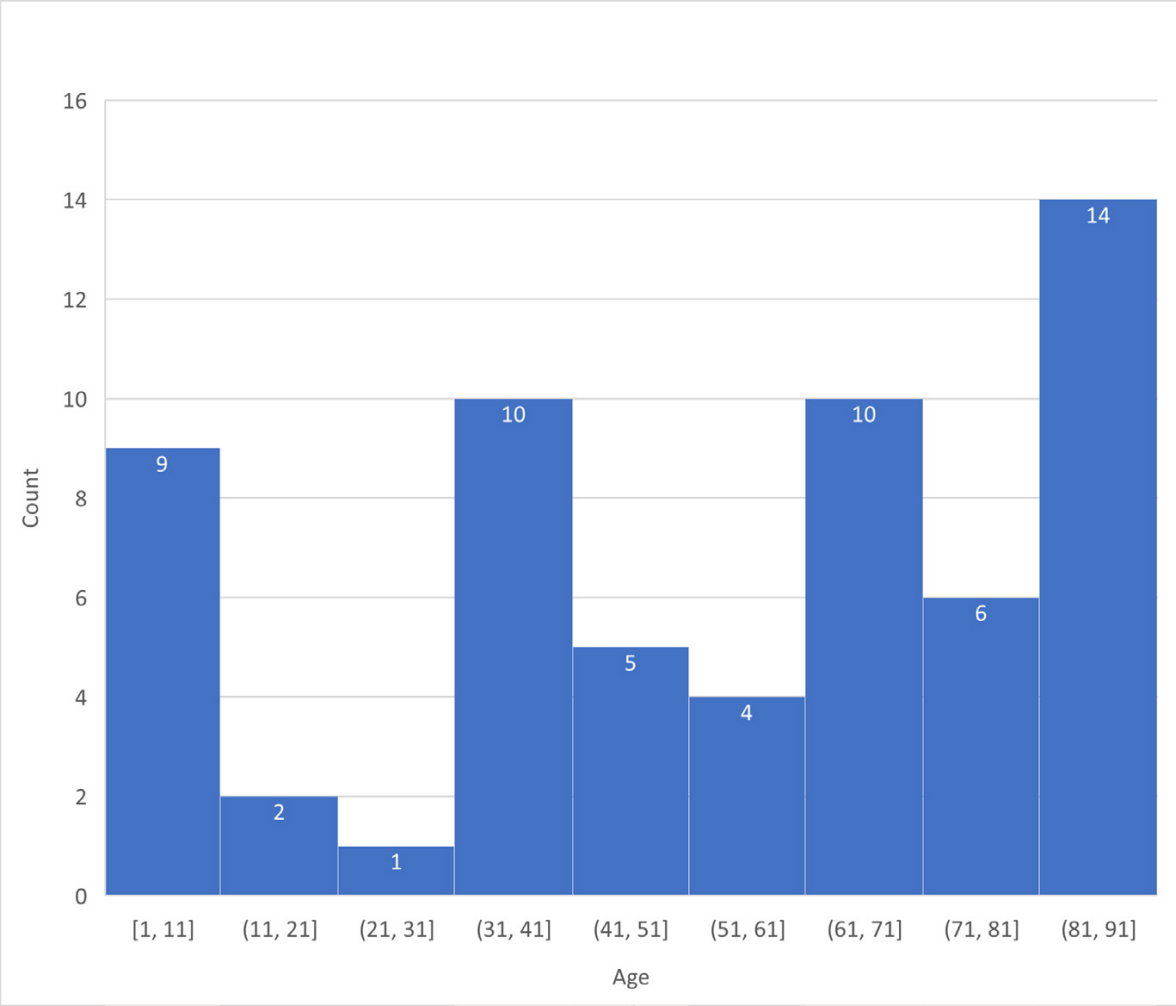
Histogram of age distribution of IPD isolates.

A total of 61 IPD blood isolates were exclusively obtained from 61 individual patients. Forty-seven isolates were collected before the COVID-19 pandemic period (2015–2019), and 14 isolates were collected during the COVID-19 pandemic period (2020–2023). Demographic factors across three age groups are shown in Table 1. The average age of the 61 patients was 53.2±28.3 years, with those aged 65 years and older representing the highest proportion at 47.5%, whereas the age group under 5 years constituted only 11.5% of the overall patient population. Most patients (82.3%) had medical conditions. Among children aged younger than 5 years, only one patient (14.3%) had pre-existing medical conditions, while over 90% of patients had pre-existing medical conditions in the other two age groups. The age distribution of IPD isolates is shown in Fig. 1. Malignancy constituted 34.4% of the pre-existing medical conditions, followed by chronic obstructive pulmonary disease (8.2%) and human immunodeficiency virus infection with stage of acquired immunodeficiency syndrome (AIDS) (6.6%). Among patients with comorbidities of malignancy, head and neck cancer accounted for 42.9% of all cancer patients.

**Table 1.** Demographic information of 61 IPD cases

Characteristic	Value
Age mean±sd (years)	53.2±28.3
Age <5 years, *N* (%)	7 (11.5)
Age 5–64 years, *N* (%)	25 (41.0)
Age ≥65 years, *N* (%)	29 (47.5)
Male	39 (63.9)
Pre-existing conditions, *N* (%)	52 (82.3)
Age <5 years, *N* (%)	1 (14.3)
Age 5–64 years, *N* (%)	23 (92.0)
Age ≥65 years, *N* (%)	28 (96.6)
Malignancy, *N* (%)	21 (34.43)
Head and neck cancer, *N* (%)	11 (18.0)
Nasopharyngeal cancer, *N* (%)	4 (6.6)
Tongue cancer (squamous cell carcinoma), *N* (%)	1 (1.6)
Parotid carcinoma, *N* (%)	1 (1.6)
Lung cancer with gingiva involvement, *N* (%)	1 (1.6)
Oesophageal cancer, *N* (%)	1 (1.6)
Multiple myeloma with buccal mucosa involvement, *N* (%)	1 (1.6)
Pituitary macroadenoma	1 (1.6)
Other malignancies	10 (16.4)
Gastric cancer	2 (3.2)
Lymphoma	1 (1.6)
Renal cell carcinoma	1 (1.6)
Multiple myeloma	1 (1.6)
Mixed adenoneuroendocrine carcinoma	1 (1.6)
Lung cancer	1 (1.6)
Signet ring cell carcinoma of the stomach	1 (1.6)
Gastrointestinal stromal tumour	1 (1.6)
Colon cancer	1 (1.6)
COPD, *N* (%)	5 (8.2)
HIV infection with AIDS status, *N* (%)	4 (6.6)
Others, *N* (%)	31 (51)
PCV13 serotypes coverage rate, *N* (%)	32 (52.5)
Age <5 years, *N* (%)	3 (42.9)
Age 5–64 years, *N* (%)	15 (60.0)
Age ≥65 years, *N* (%)	11 (37.9)
Serotype 19A	
Age <5 years, *N* (%)	3 (25.0)
Age 5–64 years, *N* (%)	6 (50.0)
Age ≥65 years, *N* (%)	3 (25.0)

COPD, chronic obstructive pulmonary disease; HIV, human immunodeficiency virus.

### Serotype distributions of IPD isolates

Serotype 19A was the most frequently identified serotype, found in 12 (19.7%) isolates. The second most frequent serotypes were 3, 23A and 23F, with three isolates (4.9%) each. The serotype of one isolate was not determined due to the serotype sequence target not being found. The remaining pneumococcal serotypes are listed in Table 2. Overall, the coverage rate of PCV13 serotypes was 52.5%, and the coverage of PPSV23 was 62.3%. Patients aged 65 years and older exhibited the lowest coverage rate at 37.9%.

**Table 2.** Serotype distribution of the 61 pneumococcal strains

Pneumococcal serotypes, *N* (%)							
**PCV13**	32 (52.5)	**Non-PCV13**	29 (47.5)	**PPSV23**	38 (62.3)	**Non-PPSV23**	23 (37.7)
19A	12 (19.7)	23A	5 (8.2)	19A	12 (19.7)	23A	5 (8.2)
3	5 (8.2)	34	4 (6.7)	3	5 (8.2)	34	4 (6.7)
23F	5 (8.2)	15A	4 (6.7)	23F	5 (8.2)	15A	4 (6.7)
14	4 (6.7)	15B	4 (6.7)	14	4 (6.7)	15C	3 (4.9)
19F	3 (4.9)	15C	3 (4.9)	15B	4 (6.7)	35B	3 (4.9)
6B	2 (3.3)	35B	3 (4.9)	19F	3 (4.9)	13	1 (1.6)
6A	1 (1.6)	8	1 (1.6)	6B	2 (3.3)	22A	1 (1.6)
		11A	1 (1.6)	20	1 (1.6)	6A	1 (1.6)
		13	1 (1.6)	8	1 (1.6)	NF	1 (1.6)
		20	1 (1.6)	11A	1 (1.6)		
		22A	1 (1.6)				
		NF	1 (1.6)				

NF: Not found

### Antibiotic resistance

Table 3 shows the percentage of non-susceptible isolates among the 61 IPD isolates in this study. Among the 61 isolates, the proportion of penicillin-non-susceptible strains was 26.2% based on the non-meningitis breakpoint and 85.2% based on the meningitis breakpoint. PCV serotypes had a significantly higher rate of penicillin nonsusceptibility compared to non-PCV13 serotypes. The rates of cefotaxime nonsusceptibility were 59.0% using the meningitis breakpoint and 24.6% using the non-meningitis breakpoint. Using the non-meningitis breakpoint, PCV13 serotypes exhibited a significantly higher rate of cefotaxime non-susceptibility compared with non-PCV13 serotypes (40.6% vs. 6.9%). Notably, 43 isolates (70.5%) were predicted by WGS to be non-susceptible to meropenem, including 16 resistant isolates (MIC=1 µg ml^−1^) and 27 intermediate isolates (MIC=0.5 µg ml^−1^). Most of these meropenem-resistant isolates (12 out of 16 isolates) were serotype 19A/ST320. Resistance to erythromycin was observed in 86.9% of all isolates. Table 3 also shows that all 12 isolates of serotype 19A were non-susceptible to penicillin, cefotaxime and meropenem. Table 4 details the non-susceptibility patterns of isolates to various antibiotics, stratified by age, cancer status and time of isolation with regard to the COVID-19 pandemic. Differences between the demographic groups were mostly insignificant. Notably, levofloxacin non-susceptible isolates were more frequently observed in patients aged ≥65 years (5/29) compared to those aged <65 years (0/32; *P*=0.02).

**Table 3.** Percentage of non-susceptible isolates of *S. pneumoniae* including all serotypes and stratified by PCV13 serotypes

Antibiotics, *N* (%)		Total	PCV13 *N*=**32**	Non PCV13 *N*=**29**	*P* value	Serotype 19**A**
PCN	Meningitis	52 (85.2)	27 (84.4)	25 (86.2)	1.0	12 (100.0)
	Non-meningitis	16 (26.2)	14 (43.8)	2 (6.9)	0.001	12 (100.0)
AMO		22 (36.1)	14 (43.8)	8 (27.6)	0.29	12 (100.0)
TAX	Meningitis	36 (59.0)	22 (68.8)	14 (48.3)	0.12	12 (100.0)
	Non-meningitis	15 (24.6)	13 (40.6)	2 (6.9)	0.003	12 (100.0)
CFX		45 (73.8)	25 (78.1)	20 (69.0)	0.56	12 (100.0)
MER		43 (70.5)	23 (71.9)	20 (69.0)	1.0	12 (100.0)
VAN		0 (0.0)	0 (0.0)	0 (0.0)	1.0	0 (0.0)
LFX		5 (8.2)	4 (12.5)	1 (3.5)	0.36	0 (0.0)
ERY		53 (86.9)	29 (90.6)	24 (82.8)	0.46	12 (100.0)
CLI		49 (80.3)	27 (84.4)	22 (75.9)	0.52	12 (100.0)
TET		55 (90.2)	31 (96.9)	24 (82.8)	0.09	12 (100.0)
COT		37 (60.7)	26 (81.3)	11 (37.9)	0.0007	12 (100.0)
CHL		12 (19.7)	6 (18.8)	6 (20.7)	1.0	0 (0.0)
RIF		0 (0.0)	0 (0.0)	0 (0.0)	1.0	0 (0.0)
LZO		0 (0.0)	0 (0.0)	0 (0.0)	1.0	0 (0.0)

*P* values represent the comparison between PCV13 and non-PCV13 groups using Fisher’s exact test.

AMO, amoxicillin; CFX, ceftriaxone; CHL, chloramphenicol; CLI, clindamycin; COT, trimethoprim–sulfamethoxazole; ERY, erythromycin; LFX, levofloxacin; LZO, linezolid; MER, meropenem; MER, meropenem; PCN, penicillin; RIF, rifampin; TAX, cefotaxime; TET, tetracyclin; VAN, vancomycin.

**Table 4.** Percentage of non-susceptible isolates of *S. pneumoniae* including all serotypes and stratified by age of 65, pre-existing medical condition of cancer and period of isolation based on the COVID-19 pandemic

	Age			Cancer			Pandemic		
Antibiotics, *N* (%)	≥65	<65	*P* value	Yes	No	*P* value	Pre-pandemic	Post-pandemic	*P* value
PCN									
Meningitis	26 (89.7)	26 (81.3)	0.48	20 (95.2)	32 (80.0)	0.15	40 (85.1)	12 (85.7)	1.0
Non-meningitis	6 (20.7)	10 (31.3)	0.40	8 (38.1)	8 (20.0)	0.14	11 (23.4)	5 (35.7)	0.49
AMO	10 (34.5)	12 (37.5)	1.0	10 (47.6)	12 (30.0)	0.26	15 (31.9)	7 (50.0)	0.34
TAX									
Meningitis	19 (65.5)	17 (53.1)	0.43	16 (76.2)	20 (50.0)	0.06	24 (51.1)	12 (85.7)	0.03
Non-meningitis	5 (17.2)	10 (31.3)	0.24	7 (33.3)	8 (20.0)	0.35	11 (23.1)	4 (28.6)	0.73
CFX	23 (79.3)	22 (68.8)	0.40	18 (85.7)	27 (67.5)	0.22	33 (70.2)	12 (85.7)	0.32
MER	23 (79.3)	20 (62.5)	0.17	18 (85.7)	25 (62.5)	0.08	31 (66.0)	12 (85.7)	0.20
VAN	0 (0.0)	0 (0.0)		0 (0.0)	0 (0.0)		0 (0.0)	0 (0.0)	
LFX	5 (17.2)	0 (0)	0.02	2 (9.5)	3 (7.5)	1.0	2 (4.3)	3 (21.4)	0.07
RIF	0 (0.0)	0 (0.0)		0 (0.0)	0 (0.0)		0 (0.0)	0 (0.0)	
LZO	0 (0.0)	0 (0.0)		0 (0.0)	0 (0.0)		0 (0.0)	0 (0.0)	

AMO, amoxicillin; CFX, ceftriaxone; LFX, levofloxacin; LZO, linezolid; MER, meropenem; MER, meropenem; PCN, penicillin; RIF, rifampin; TAX, cefotaxime; VAN, vancomycin.

### Phylogenetic analysis

**Fig. 2.**
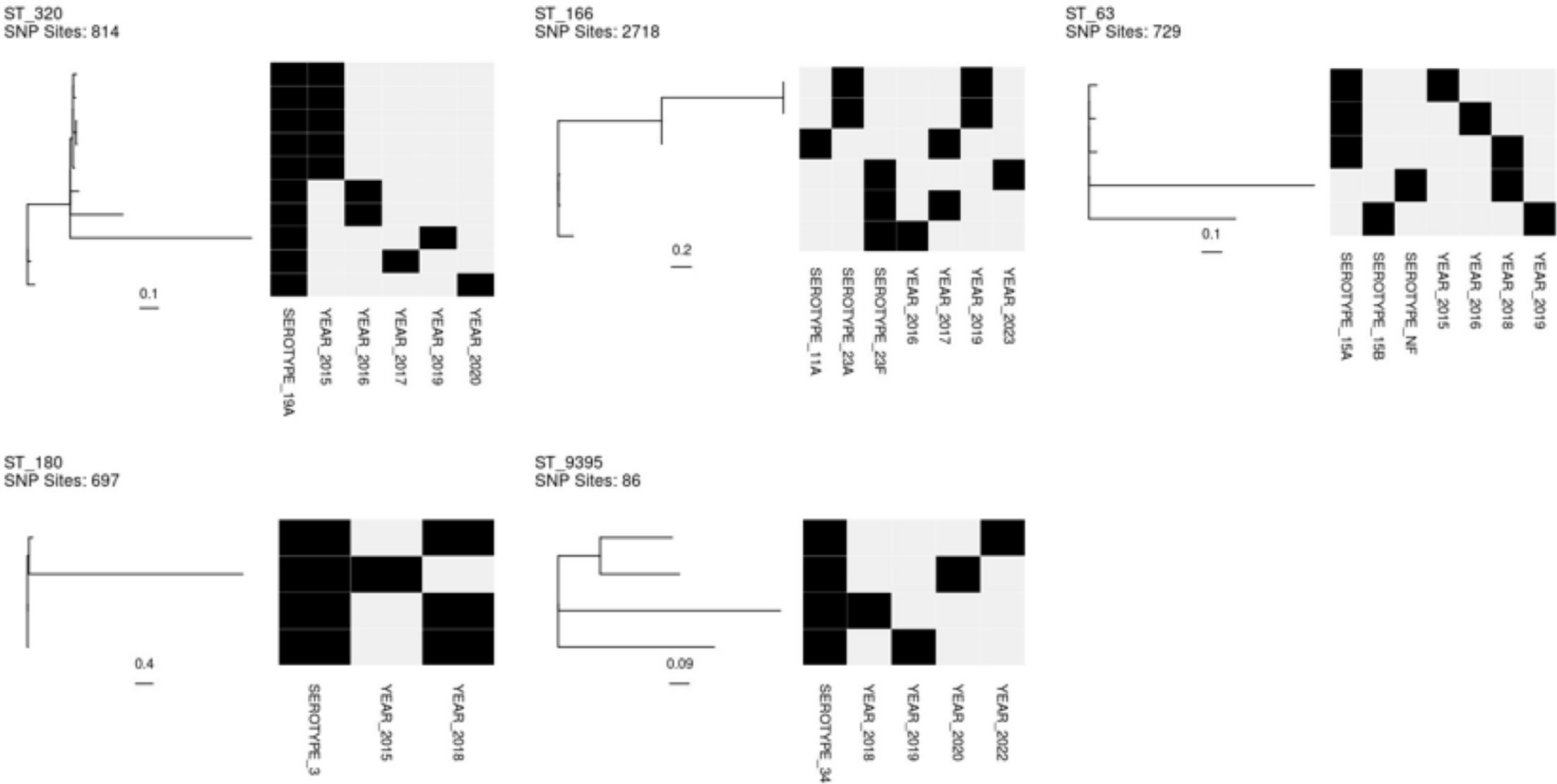
Phylogenetic analysis by ST. For each ST, core-genome SNPs were identified and used to construct the tree using the kSNP software. Length of the core-genome SNP alignment (SNP sites) is shown. Scale bar indicates expected nucleotide substitutions per site. The serotype and sample years of each isolate are shown in black blocks.

A total of 24 STs were identified in this study, among which 5 had 4 or more isolates: ST320 (*n*=10), ST166 (*n*=6), ST63 (*n*=5), ST180 (*n*=4) and ST9395 (*n*=4). For each of the five major STs, a phylogenetic tree was constructed to illustrate evolutionary relationships among IPD isolates within the lineage (Fig. 2). There was a substantial variation of nucleotide diversity across the five lineages. In particular, the pair-wise SNP distance among ST9395 isolates (112±31 SNPs, mean±sd) was significantly smaller than that among isolates within the other four lineages (1,061±1,711 SNPs; *P*<0.001, t-test), consistent with ST9395 emerging from a more recent ancestor. All four ST9395 isolates were serotype 34 (non-PCV13 serotype) and non-susceptible to penicillin (meningitis breakpoint) and meropenem. One ST9395 isolate was also resistant to erythromycin, tetracycline and chloramphenicol.

## Discussion

Compared with the pre-pandemic period (*n*=47), substantially fewer isolates were collected during the COVID-19 pandemic (*n*=14). Numerous studies from different countries have shown that the implementation of social-distancing measures during the COVID-19 pandemic led to marked declines in respiratory viral infections [22]. Because antecedent viral infections play an important role in predisposing individuals to IPD, the reduction in viral circulation likely contributed to the observed decrease in IPD incidence. In Taiwan, social-restrictive policies were implemented during the COVID-19 pandemic, and the incidence of several respiratory viruses declined substantially during this period [23]. Our findings are consistent with these previously reported epidemiological patterns. In addition, reduced healthcare-seeking behaviour during the pandemic may have further contributed to the decline in reported IPD cases [23,24].

The age distribution of IPD in our study showed that 11.5% of IPD isolates were obtained from children aged ≤5 years in the post-PCV13 era. According to the National Notifiable Disease Surveillance Report from the Taiwan Centers for Disease Control, the proportion of IPD cases among children aged ≤5 years declined from 19.7% in 2013 before the introduction of PCV13 to 8.0% in 2024 [25,26]. The slightly higher proportion observed in our cohort likely reflects inclusion of earlier post-PCV13 years, before the full population-level impact of vaccination was realized. Accordingly, age-specific differences in PCV13 serotype distribution were less pronounced in our study.

In our study, the serotypes included in PCV13 accounted for 52.5% of our IPD isolates. Additionally, serotype 19A, one of the PCV13 vaccine types (VTs), was the leading IPD serotype and represented 19.7% of total isolates. Although serotype 19A remained the dominant serotype in our study population, which consisted predominantly of adult IPD cases, the referenced study indicates that serotype 19A is no longer the leading serotype among children under 5 years of age in Taiwan following PCV13 implementation [8]. Among the general population in Taiwan, serotype replacement following the implementation of PCV13 has been observed from 2015 through 2020, with serotypes 15A and 23A overtaking 19A as the most common IPD serotypes among all age groups [10,11]. Two studies from Taiwan reported that PCV13 serotypes accounted for 31.5% and 21.1% of IPD cases in the general population in 2018 and 2022, respectively [11,27]. In contrast, the proportion of PCV13 serotypes in our study was higher (51.2%). This discrepancy may be attributable to the longer study period in our analysis, which spanned 8 years (2015–2023). PCV13 was incorporated into Taiwan’s national immunization programme in 2015, and the incidence of IPD caused by PCV13 serotypes remained relatively high during the early years following vaccine implementation. Exception for serotype 3, numerous studies from various countries have reported substantial indirect protection against PCV13 serotypes in the post-PCV13 era [28]. However, the patterns of serotype replacement following the implementation of PCVs have diverged across different countries [29]. There remains a burden of IPD caused by PCV13 VTs, such as 19A, 7F and 14 in some countries [28,30, 31]. The 20-valent pneumococcal conjugate vaccine (PCV20) has been proposed for future incorporation into Taiwan’s NIP. With the inclusion of the seven additional serotypes beyond those covered by PCV13, the estimated serotype coverage in our dataset would increase from 52.5% with PCV15 to 62.3% with PCV20. Continued pneumococcal serotype surveillance will be essential to monitor potential serotype replacement following PCV20 implementation in Taiwan.

Patients under 5 years old made up 11.5% of the total number of isolates in this study, and patients between 5 and 64 years old and 65 years or older made up 41.0% and 47.5%, respectively. The age distribution of our study was severely skewed towards the adult population, which is consistent with the current age distribution of IPD in Taiwan [25]. Almost all isolates from adults in our study were from patients with pre-existing medical conditions, with malignancy being the most common comorbidity (34.3%). Previous studies have demonstrated that the incidence of IPD among patients with malignancy is significantly higher than that in the general population [32,36]. In particular, individuals with haematological malignancies and solid tumours are at especially high risk [32,34, 36]. In our study, IPD cases involving patients with head and neck tumours accounted for nearly half (9 out of 21) of the malignancy-associated cases. Given the limited sample size, this finding should be interpreted with caution and requires validation in larger studies. While we found only one study supporting this observation, it did not explain the pathogenesis linking head and neck tumours to IPD [37]. We speculate that disruption of the nasopharyngeal epithelium caused by tumours or radiotherapy may contribute to this association. Consequently, patients may benefit from receiving pneumococcal vaccines as soon as a cancer diagnosis is made, preferably before initiating radiotherapy or chemotherapy [37,40].

We found that 70.5% of isolates in our study were non-susceptible to meropenem across both vaccine and non-vaccine serotypes. Two studies from Taiwan have previously investigated the prevalence of meropenem non-susceptibility among pneumococcal isolates, reporting rates of 60.7% during 2013–2017 and 70.8% in 2018, respectively [17,41]. The meropenem non-susceptibility rate observed in our study is, therefore, comparable to these earlier findings. These previous studies also documented the emergence of meropenem-non-susceptible pneumococcal strains, including serotype–ST combinations such as 15A-ST63, 15B/C-ST83 and 23A-ST166. Meropenem is not a first-line antibiotic for the treatment of pneumococcal infections. In clinical practice, particularly in the setting of severe or emergent infectious diseases, meropenem is often administered empirically as a broad-spectrum agent to provide coverage for potential Gram-negative pathogens. However, not all emergent infections are caused by Gram-negative bacteria; consequently, reliance on meropenem alone may be inadequate for the treatment of acute pneumococcal infections. Although meropenem is indicated for certain pneumococcal infections, such as bacterial meningitis, most clinical microbiology laboratories do not routinely screen for meropenem susceptibility in *S. pneumoniae*, which may pose potential challenges in clinical management. To date, few studies have specifically addressed meropenem non-susceptibility rates in pneumococcal isolates. In this study, phylogenetic analysis suggested the recent emergence of meropenem-non-susceptible serotype 34/ST9395 isolates, highlighting the importance of continuous antimicrobial resistance monitoring through genomic surveillance. In our study, the resistance rate of levofloxacin is significantly higher among those equal to or older than 65 years old. Few studies investigated the association between age and fluoroquinolone resistance rate to pneumococcus. One study indicated that the elderly population has more antibiotic exposure, which can select resistant strains [42].

This study had several limitations. First, the observed IPD cases may not fully represent the true incidence trends of invasive pneumococcal disease in southern Taiwan, as this study was conducted at a single medical centre in a region served by three tertiary medical centres. In addition, the annual number of IPD cases in Taiwan declined substantially following the introduction of the PCV13 into the national immunization programme for children under 5 years of age, which has made large-scale IPD investigations increasingly difficult. Second, some clinical information, such as individual vaccination status and clinical outcome data, was not included in this study. Third, the number of isolates included in this study was relatively small, particularly during 2020–2023, when the incidence of respiratory tract infections other than COVID-19 declined markedly due to non-pharmaceutical interventions. Finally, the findings may have been influenced by local clinical practices, healthcare resources and diagnostic capacity. Therefore, caution is warranted when generalizing these results.

In conclusion, this study provides preliminary evidence suggesting that older adults and individuals with pre-existing medical conditions, particularly malignancy, may benefit from targeted prevention efforts. Given the markedly reduced incidence of IPD in Taiwan in recent years, multi-centre collaboration will be necessary to achieve sufficient sample sizes for large-scale analyses. Continued surveillance of serotype replacement and antimicrobial resistance remains critical for informing future pneumococcal conjugate vaccine implementation strategies.

## Abbreviations

AIDS: acquired immunodeficiency syndrome
AST: antimicrobial susceptibility
IPD: invasive pneumococcal disease
MICs: minimum inhibitory concentrations
PBP: penicillin-binding protein
PCV7: seven-valent pneumococcal conjugate vaccine
PCV13: 13-valent pneumococcal conjugate vaccine
PCV20: 20-valent pneumococcal conjugate vaccine
ST: sequence type
VTs: vaccine types
WGS: whole-genome sequencing
